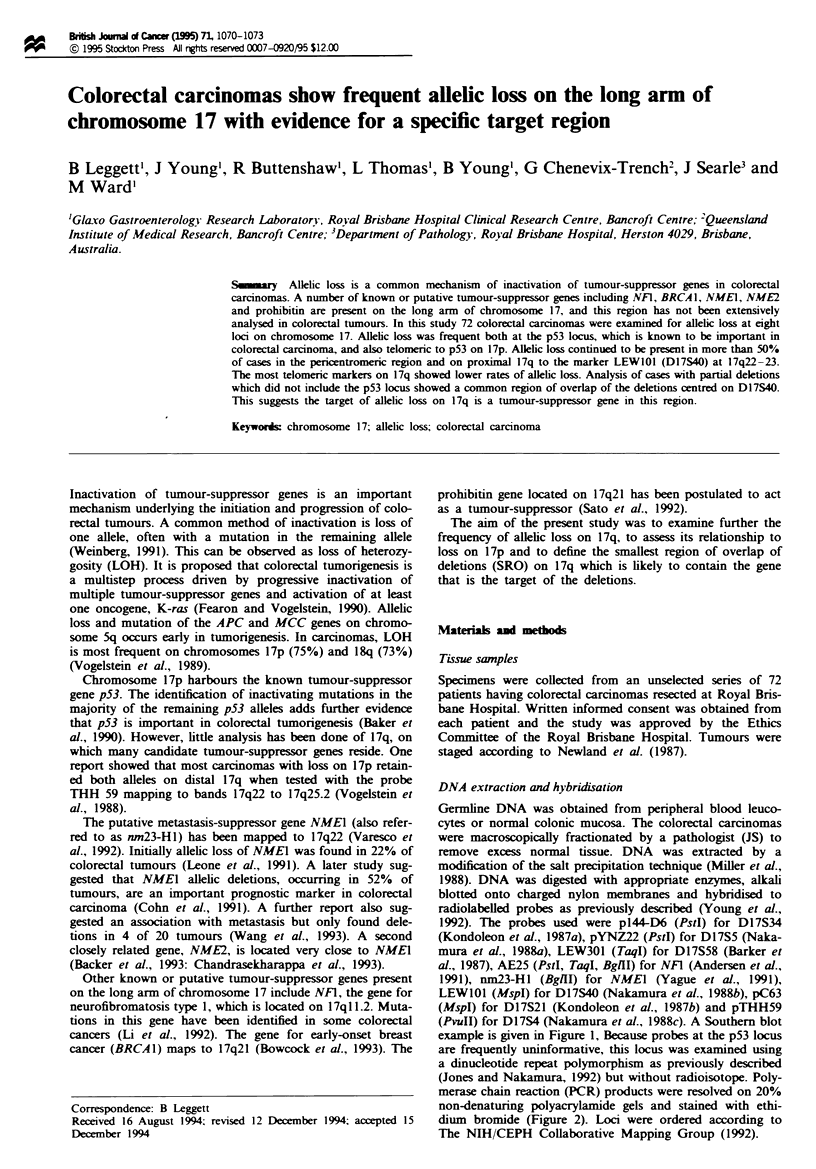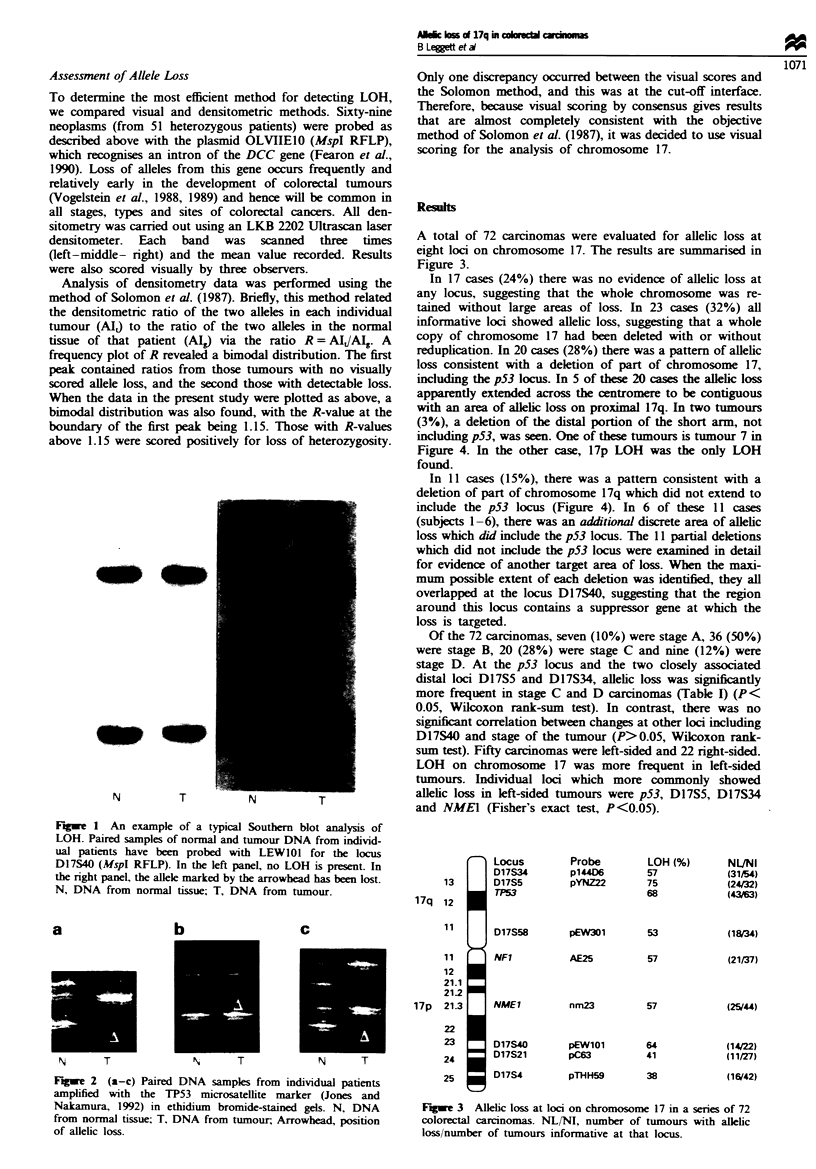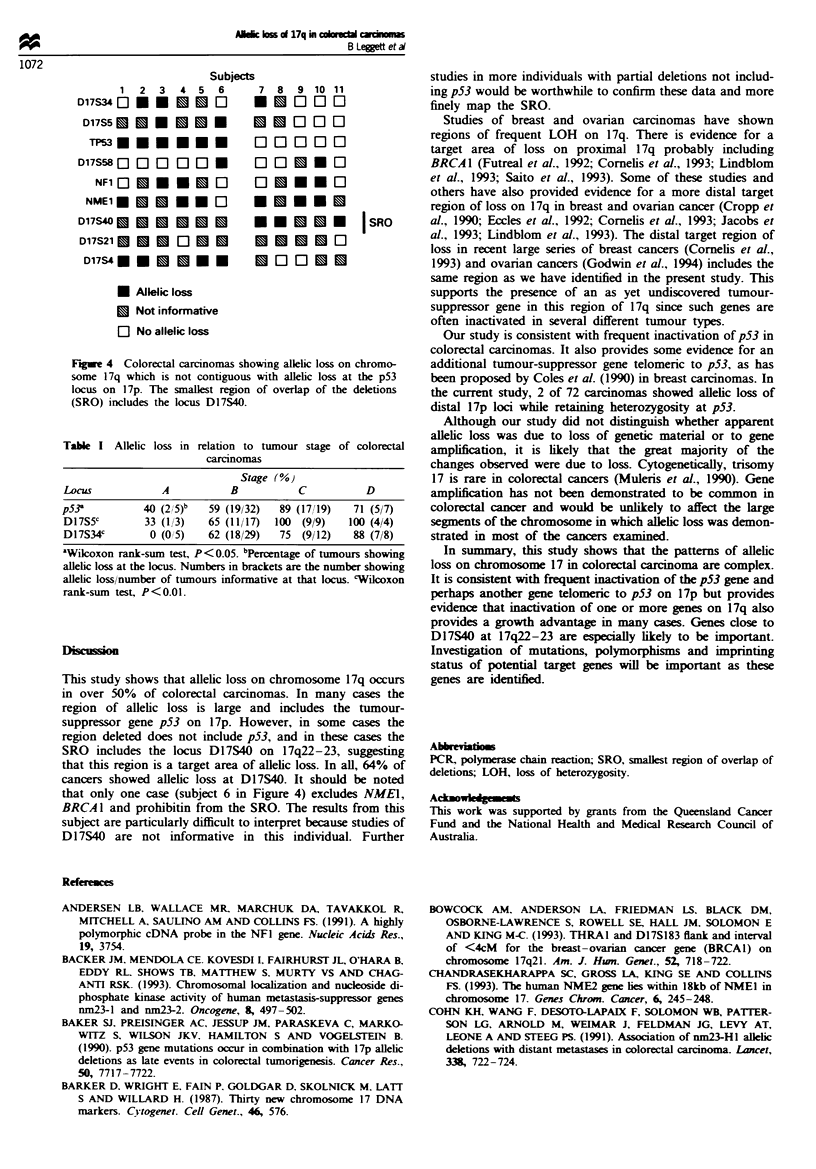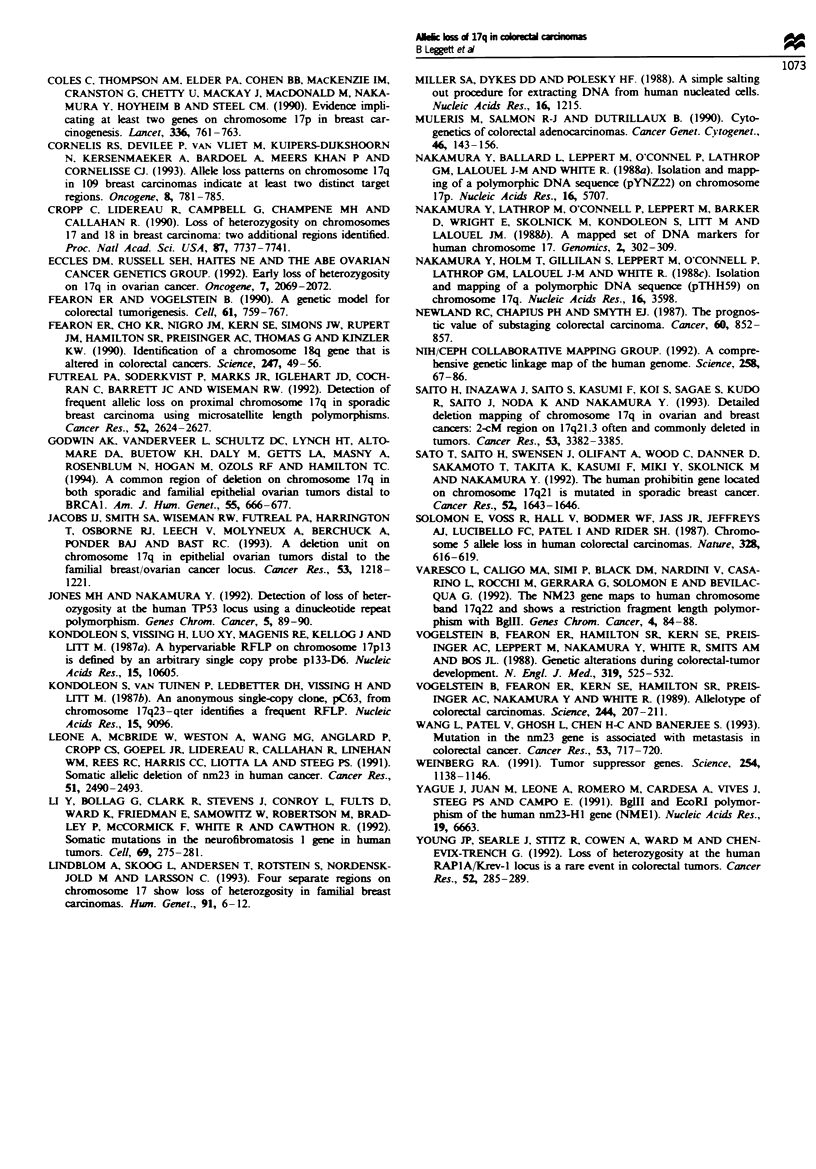# Colorectal carcinomas show frequent allelic loss on the long arm of chromosome 17 with evidence for a specific target region.

**DOI:** 10.1038/bjc.1995.206

**Published:** 1995-05

**Authors:** B. Leggett, J. Young, R. Buttenshaw, L. Thomas, B. Young, G. Chenevix-Trench, J. Searle, M. Ward

**Affiliations:** Glaxo Gastroenterology Research Laboratory, Royal Brisbane Hospital Clinical Research Centre, Bancroft Centre, Australia.

## Abstract

**Images:**


British Journal of Cancer (1995) 71, 1070–1073

© 1995 Stockton Press All rights reserved 0007–0920/95 $12.00

### Colorectal carcinomas show frequent allelic loss on the long arm of chromosome 17 with evidence for a specific target region

B Leggett[1], J Young[1], R Buttenshaw[1], L Thomas[1], B Young[1], G Chenevix-Trench[2], J Searle[3] and M Ward[1]

[1]*Glaxo Gastroenterology Research Laboratory, Royal Brisbane Hospital Clinical Research Centre, Bancroft Centre;* [2]*Queensland Institute of Medical Research, Bancroft Centre;* [3]*Department of Pathology, Royal Brisbane Hospital, Herston 4029, Brisbane, Australia.*

**Summary** Allelic loss is a common mechanism of inactivation of tumour-suppressor genes in colorectal carcinomas. A number of known or putative tumour-suppressor genes including *NF*1, *BRCA*1, *NME*1, *NME*2 and prohibitin are present on the long arm of chromosome 17, and this region has not been extensively analysed in colorectal tumours. In this study 72 colorectal carcinomas were examined for allelic loss at eight loci on chromosome 17. Allelic loss was frequent both at the p53 locus, which is known to be important in colorectal carcinoma, and also telomeric to p53 on 17p. Allelic loss continued to be present in more than 50% of cases in the pericentromeric region and on proximal 17q to the marker LEW101 (D17S40) at 17q22–23. The most telomeric markers on 17q showed lower rates of allelic loss. Analysis of cases with partial deletions which did not include the p53 locus showed a common region of overlap of the deletions centred on D17S40. This suggests the target of allelic loss on 17q is a tumour-suppressor gene in this region.

**Keywords:** chromosome 17; allelic loss; colorectal carcinoma

Inactivation of tumour-suppressor genes is an important mechanism underlying the initiation and progression of colorectal tumours. A common method of inactivation is loss of one allele, often with a mutation in the remaining allele (Weinberg, 1991). This can be observed as loss of heterozygosity (LOH). It is proposed that colorectal tumorigenesis is a multistep process driven by progressive inactivation of multiple tumour-suppressor genes and activation of at least one oncogene, K-*ras* (Fearon and Vogelstein, 1990). Allelic loss and mutation of the *APC* and *MCC* genes on chromosome 5q occurs early in tumorigenesis. In carcinomas, LOH is most frequent on chromosomes 17p (75%) and 18q (73%) (Vogelstein *et al.*, 1989).

Chromosome 17p harbours the known tumour-suppressor gene *p53*. The identification of inactivating mutations in the majority of the remaining *p53* alleles adds further evidence that *p53* is important in colorectal tumorigenesis (Baker *et al.*, 1990). However, little analysis has been done of 17q, on which many candidate tumour-suppressor genes reside. One report showed that most carcinomas with loss on 17p retained both alleles on distal 17q when tested with the probe THH 59 mapping to bands 17q22 to 17q25.2 (Vogelstein *et al.*, 1988).

The putative metastasis-suppressor gene *NME*1 (also referred to as *nm*23-H1) has been mapped to 17q22 (Varesco *et al.*, 1992). Initially allelic loss of *NME*1 was found in 22% of colorectal tumours (Leone *et al.*, 1991). A later study suggested that *NME*1 allelic deletions, occurring in 52% of tumours, are an important prognostic marker in colorectal carcinoma (Cohn *et al.*, 1991). A further report also suggested an association with metastasis but only found deletions in 4 of 20 tumours (Wang *et al.*, 1993). A second closely related gene, *NME*2, is located very close to *NME*1 (Backer *et al.*, 1993: Chandrasekharappa *et al.*, 1993).

Other known or putative tumour-suppressor genes present on the long arm of chromosome 17 include *NF*1, the gene for neurofibromatosis type 1, which is located on 17q11.2. Mutations in this gene have been identified in some colorectal cancers (Li *et al.*, 1992). The gene for early-onset breast cancer (*BRCA*1) maps to 17q21 (Bowcock *et al.*, 1993). The prohibitin gene located on 17q21 has been postulated to act as a tumour-suppressor (Sato *et al.*, 1992).

The aim of the present study was to examine further the frequency of allelic loss on 17q, to assess its relationship to loss on 17p and to define the smallest region of overlap of deletions (SRO) on 17q which is likely to contain the gene that is the target of the deletions.

### Materials and methods

### *Tissue samples*

Specimens were collected from an unselected series of 72 patients having colorectal carcinomas resected at Royal Brisbane Hospital. Written informed consent was obtained from each patient and the study was approved by the Ethics Committee of the Royal Brisbane Hospital. Tumours were staged according to Newland *et al.* (1987).

### *DNA extraction and hybridisation*

Germline DNA was obtained from peripheral blood leucocytes or normal colonic mucosa. The colorectal carcinomas were macroscopically fractionated by a pathologist (JS) to remove excess normal tissue. DNA was extracted by a modification of the salt precipitation technique (Miller *et al.*, 1988). DNA was digested with appropriate enzymes, alkali blotted onto charged nylon membranes and hybridised to radiolabelled probes as previously described (Young *et al.*, 1992). The probes used were p144-D6 (*Pst*I) for D17S34 (Kondoleon *et al.*, 1987*a*), pYNZ22 (*Pst*I) for D17S5 (Nakamura *et al.*, 1988*a*), LEW301 (*Taq*I) for D17S58 (Barker *et al.*, 1987), AE25 (*Pst*I, *Taq*I, *Bgl*II) for *NF*1 (Andersen *et al.*, 1991), nm23-H1 (*Bgl*II) for *NME*1 (Yague *et al.*, 1991), LEW101 (*Msp*I) for D17S40 (Nakamura *et al.*, 1988*b*), pC63 (*Msp*I) for D17S21 (Kondoleon *et al.*, 1987*b*) and pTHH59 (*Pvu*II) for D17S4 (Nakamura *et al.*, 1988*c*). A Southern blot example is given in Figure 1, Because probes at the p53 locus are frequently uninformative, this locus was examined using a dinucleotide repeat polymorphism as previously described (Jones and Nakamura, 1992) but without radioisotope. Polymerase chain reaction (PCR) products were resolved on 20% non-denaturing polyacrylamide gels and stained with ethidium bromide (Figure 2). Loci were ordered according to The NIH/CEPH Collaborative Mapping Group (1992).

Correspondence: B Leggett
Received 16 August 1994; revised 12 December 1994; accepted 15 December 1994

*Assessment of Allele Loss*

To determine the most efficient method for detecting LOH, we compared visual and densitometric methods. Sixty-nine neoplasms (from 51 heterozygous patients) were probed as described above with the plasmid OLVIIE10 (*Msp*I RFLP), which recognises an intron of the *DCC* gene (Fearon *et al*., 1990). Loss of alleles from this gene occurs frequently and relatively early in the development of colorectal tumours (Vogelstein *et al*., 1988, 1989) and hence will be common in all stages, types and sites of colorectal cancers. All densitometry was carried out using an LKB 2202 Ultrascan laser densitometer. Each band was scanned three times (left–middle– right) and the mean value recorded. Results were also scored visually by three observers.

Analysis of densitometry data was performed using the method of Solomon *et al*. (1987). Briefly, this method related the densitometric ratio of the two alleles in each individual tumour ($AI_t$) to the ratio of the two alleles in the normal tissue of that patient ($AI_g$) via the ratio $R = AI_t/AI_g$. A frequency plot of $R$ revealed a bimodal distribution. The first peak contained ratios from those tumours with no visually scored allele loss, and the second those with detectable loss. When the data in the present study were plotted as above, a bimodal distribution was also found, with the $R$-value at the boundary of the first peak being 1.15. Those with $R$-values above 1.15 were scored positively for loss of heterozygosity.

**Figure 1** An example of a typical Southern blot analysis of LOH. Paired samples of normal and tumour DNA from individual patients have been probed with LEW101 for the locus D17S40 (*Msp*I RFLP). In the left panel, no LOH is present. In the right panel, the allele marked by the arrowhead has been lost. N, DNA from normal tissue; T, DNA from tumour.

**Figure 2** (a–c) Paired DNA samples from individual patients amplified with the TP53 microsatellite marker (Jones and Nakamura, 1992) in ethidium bromide-stained gels. N, DNA from normal tissue; T, DNA from tumour; Arrowhead, position of allelic loss.

Only one discrepancy occurred between the visual scores and the Solomon method, and this was at the cut-off interface. Therefore, because visual scoring by consensus gives results that are almost completely consistent with the objective method of Solomon *et al*. (1987), it was decided to use visual scoring for the analysis of chromosome 17.

### Results

A total of 72 carcinomas were evaluated for allelic loss at eight loci on chromosome 17. The results are summarised in Figure 3.

In 17 cases (24%) there was no evidence of allelic loss at any locus, suggesting that the whole chromosome was retained without large areas of loss. In 23 cases (32%) all informative loci showed allelic loss, suggesting that a whole copy of chromosome 17 had been deleted with or without reduplication. In 20 cases (28%) there was a pattern of allelic loss consistent with a deletion of part of chromosome 17, including the *p53* locus. In 5 of these 20 cases the allelic loss apparently extended across the centromere to be contiguous with an area of allelic loss on proximal 17q. In two tumours (3%), a deletion of the distal portion of the short arm, not including *p53*, was seen. One of these tumours is tumour 7 in Figure 4. In the other case, 17p LOH was the only LOH found.

In 11 cases (15%), there was a pattern consistent with a deletion of part of chromosome 17q which did not extend to include the *p53* locus (Figure 4). In 6 of these 11 cases (subjects 1–6), there was an *additional* discrete area of allelic loss which *did* include the *p53* locus. The 11 partial deletions which did not include the *p53* locus were examined in detail for evidence of another target area of loss. When the maximum possible extent of each deletion was identified, they all overlapped at the locus D17S40, suggesting that the region around this locus contains a suppressor gene at which the loss is targeted.

Of the 72 carcinomas, seven (10%) were stage A, 36 (50%) were stage B, 20 (28%) were stage C and nine (12%) were stage D. At the *p53* locus and the two closely associated distal loci D17S5 and D17S34, allelic loss was significantly more frequent in stage C and D carcinomas (Table I) ($P<0.05$, Wilcoxon rank-sum test). In contrast, there was no significant correlation between changes at other loci including D17S40 and stage of the tumour ($P>0.05$, Wilcoxon rank-sum test). Fifty carcinomas were left-sided and 22 right-sided. LOH on chromosome 17 was more frequent in left-sided tumours. Individual loci which more commonly showed allelic loss in left-sided tumours were *p53*, D17S5, D17S34 and *NME*1 (Fisher's exact test, $P<0.05$).

**Figure 3** Allelic loss at loci on chromosome 17 in a series of 72 colorectal carcinomas. NL/NI, number of tumours with allelic loss/number of tumours informative at that locus.

**Figure 4** Colorectal carcinomas showing allelic loss on chromosome 17q which is not contiguous with allelic loss at the p53 locus on 17p. The smallest region of overlap of the deletions (SRO) includes the locus D17S40.

**Table I** Allelic loss in relation to tumour stage of colorectal carcinomas

| | Stage (%) | | | |
|---|---|---|---|---|
| *Locus* | *A* | *B* | *C* | *D* |
| *p53*[a] | 40 (2/5)[b] | 59 (19/32) | 89 (17/19) | 71 (5/7) |
| D17S5[c] | 33 (1/3) | 65 (11/17) | 100 (9/9) | 100 (4/4) |
| D17S34[c] | 0 (0/5) | 62 (18/29) | 75 (9/12) | 88 (7/8) |

[a]Wilcoxon rank-sum test, $P<0.05$. [b]Percentage of tumours showing allelic loss at the locus. Numbers in brackets are the number showing allelic loss/number of tumours informative at that locus. [c]Wilcoxon rank-sum test, $P<0.01$.

### Discussion

This study shows that allelic loss on chromosome 17q occurs in over 50% of colorectal carcinomas. In many cases the region of allelic loss is large and includes the tumour-suppressor gene *p53* on 17p. However, in some cases the region deleted does not include *p53*, and in these cases the SRO includes the locus D17S40 on 17q22-23, suggesting that this region is a target area of allelic loss. In all, 64% of cancers showed allelic loss at D17S40. It should be noted that only one case (subject 6 in Figure 4) excludes *NME*1, *BRCA*1 and prohibitin from the SRO. The results from this subject are particularly difficult to interpret because studies of D17S40 are not informative in this individual. Further studies in more individuals with partial deletions not including *p53* would be worthwhile to confirm these data and more finely map the SRO.

Studies of breast and ovarian carcinomas have shown regions of frequent LOH on 17q. There is evidence for a target area of loss on proximal 17q probably including *BRCA*1 (Futreal *et al.*, 1992; Cornelis *et al.*, 1993; Lindblom *et al.*, 1993; Saito *et al.*, 1993). Some of these studies and others have also provided evidence for a more distal target region of loss on 17q in breast and ovarian cancer (Cropp *et al.*, 1990; Eccles *et al.*, 1992; Cornelis *et al.*, 1993; Jacobs *et al.*, 1993; Lindblom *et al.*, 1993). The distal target region of loss in recent large series of breast cancers (Cornelis *et al.*, 1993) and ovarian cancers (Godwin *et al.*, 1994) includes the same region as we have identified in the present study. This supports the presence of an as yet undiscovered tumour-suppressor gene in this region of 17q since such genes are often inactivated in several different tumour types.

Our study is consistent with frequent inactivation of *p53* in colorectal carcinomas. It also provides some evidence for an additional tumour-suppressor gene telomeric to *p53*, as has been proposed by Coles *et al.* (1990) in breast carcinomas. In the current study, 2 of 72 carcinomas showed allelic loss of distal 17p loci while retaining heterozygosity at *p53*.

Although our study did not distinguish whether apparent allelic loss was due to loss of genetic material or to gene amplification, it is likely that the great majority of the changes observed were due to loss. Cytogenetically, trisomy 17 is rare in colorectal cancers (Muleris *et al.*, 1990). Gene amplification has not been demonstrated to be common in colorectal cancer and would be unlikely to affect the large segments of the chromosome in which allelic loss was demonstrated in most of the cancers examined.

In summary, this study shows that the patterns of allelic loss on chromosome 17 in colorectal carcinoma are complex. It is consistent with frequent inactivation of the *p53* gene and perhaps another gene telomeric to *p53* on 17p but provides evidence that inactivation of one or more genes on 17q also provides a growth advantage in many cases. Genes close to D17S40 at 17q22-23 are especially likely to be important. Investigation of mutations, polymorphisms and imprinting status of potential target genes will be important as these genes are identified.

### Abbreviations

PCR, polymerase chain reaction; SRO, smallest region of overlap of deletions; LOH, loss of heterozygosity.

### Acknowledgements

This work was supported by grants from the Queensland Cancer Fund and the National Health and Medical Research Council of Australia.

### References

ANDERSEN LB, WALLACE MR, MARCHUK DA, TAVAKKOL R, MITCHELL A, SAULINO AM AND COLLINS FS. (1991). A highly polymorphic cDNA probe in the NF1 gene. *Nucleic Acids Res.*, **19**, 3754.

BACKER JM, MENDOLA CE, KOVESDI I, FAIRHURST JL, O'HARA B, EDDY RL, SHOWS TB, MATTHEW S, MURTY VS AND CHAGANTI RSK. (1993). Chromosomal localization and nucleoside diphosphate kinase activity of human metastasis-suppressor genes nm23-1 and nm23-2. *Oncogene*, **8**, 497-502.

BAKER SJ, PREISINGER AC, JESSUP JM, PARASKEVA C, MARKOWITZ S, WILSON JKV, HAMILTON S AND VOGELSTEIN B. (1990). p53 gene mutations occur in combination with 17p allelic deletions as late events in colorectal tumorigenesis. *Cancer Res.*, **50**, 7717-7722.

BARKER D, WRIGHT E, FAIN P, GOLDGAR D, SKOLNICK M, LATT S AND WILLARD H. (1987). Thirty new chromosome 17 DNA markers. *Cytogenet. Cell Genet.*, **46**, 576.

BOWCOCK AM, ANDERSON LA, FRIEDMAN LS, BLACK DM, OSBORNE-LAWRENCE S, ROWELL SE, HALL JM, SOLOMON E AND KING M-C. (1993). THRA1 and D17S183 flank and interval of <4cM for the breast-ovarian cancer gene (BRCA1) on chromosome 17q21. *Am. J. Hum. Genet.*, **52**, 718-722.

CHANDRASEKHARAPPA SC, GROSS LA, KING SE AND COLLINS FS. (1993). The human NME2 gene lies within 18kb of NME1 in chromosome 17. *Genes Chrom. Cancer*, **6**, 245-248.

COHN KH, WANG F, DESOTO-LAPAIX F, SOLOMON WB, PATTERSON LG, ARNOLD M, WEIMAR J, FELDMAN JG, LEVY AT, LEONE A AND STEEG PS. (1991). Association of nm23-H1 allelic deletions with distant metastases in colorectal carcinoma. *Lancet*, **338**, 722-724.

COLES C, THOMPSON AM, ELDER PA, COHEN BB, MACKENZIE IM, CRANSTON G, CHETTY U, MACKAY J, MACDONALD M, NAKAMURA Y, HOYHEIM B AND STEEL CM. (1990). Evidence implicating at least two genes on chromosome 17p in breast carcinogenesis. *Lancet*, **336**, 761-763.

CORNELIS RS, DEVILEE P, VAN VLIET M, KUIPERS-DIJKSHOORN N, KERSENMAEKER A, BARDOEL A, MEERS KHAN P AND CORNELISSE CJ. (1993). Allele loss patterns on chromosome 17q in 109 breast carcinomas indicate at least two distinct target regions. *Oncogene*, **8**, 781-785.

CROPP C, LIDEREAU R, CAMPBELL G, CHAMPENE MH AND CALLAHAN R. (1990). Loss of heterozygosity on chromosomes 17 and 18 in breast carcinoma: two additional regions identified. *Proc. Natl Acad. Sci. USA*, **87**, 7737-7741.

ECCLES DM, RUSSELL SEH, HAITES NE AND THE ABE OVARIAN CANCER GENETICS GROUP. (1992). Early loss of heterozygosity on 17q in ovarian cancer. *Oncogene*, **7**, 2069-2072.

FEARON ER AND VOGELSTEIN B. (1990). A genetic model for colorectal tumorigenesis. *Cell*, **61**, 759-767.

FEARON ER, CHO KR, NIGRO JM, KERN SE, SIMONS JW, RUPERT JM, HAMILTON SR, PREISINGER AC, THOMAS G AND KINZLER KW. (1990). Identification of a chromosome 18q gene that is altered in colorectal cancers. *Science*, **247**, 49-56.

FUTREAL PA, SODERKVIST P, MARKS JR, IGLEHART JD, COCHRAN C, BARRETT JC AND WISEMAN RW. (1992). Detection of frequent allelic loss on proximal chromosome 17q in sporadic breast carcinoma using microsatellite length polymorphisms. *Cancer Res.*, **52**, 2624-2627.

GODWIN AK, VANDERVEER L, SCHULTZ DC, LYNCH HT, ALTOMARE DA, BUETOW KH, DALY M, GETTS LA, MASNY A, ROSENBLUM N, HOGAN M, OZOLS RF AND HAMILTON TC. (1994). A common region of deletion on chromosome 17q in both sporadic and familial epithelial ovarian tumors distal to BRCA1. *Am. J. Hum. Genet.*, **55**, 666-677.

JACOBS IJ, SMITH SA, WISEMAN RW, FUTREAL PA, HARRINGTON T, OSBORNE RJ, LEECH V, MOLYNEUX A, BERCHUCK A, PONDER BAJ AND BAST RC. (1993). A deletion unit on chromosome 17q in epithelial ovarian tumors distal to the familial breast/ovarian cancer locus. *Cancer Res.*, **53**, 1218-1221.

JONES MH AND NAKAMURA Y. (1992). Detection of loss of heterozygosity at the human TP53 locus using a dinucleotide repeat polymorphism. *Genes Chrom. Cancer*, **5**, 89-90.

KONDOLEON S, VISSING H, LUO XY, MAGENIS RE, KELLOG J AND LITT M. (1987*a*). A hypervariable RFLP on chromosome 17p13 is defined by an arbitrary single copy probe p133-D6. *Nucleic Acids Res.*, **15**, 10605.

KONDOLEON S, VAN TUINEN P, LEDBETTER DH, VISSING H AND LITT M. (1987*b*). An anonymous single-copy clone, pC63, from chromosome 17q23-qter identifies a frequent RFLP. *Nucleic Acids Res.*, **15**, 9096.

LEONE A, MCBRIDE W, WESTON A, WANG MG, ANGLARD P, CROPP CS, GOEPEL JR, LIDEREAU R, CALLAHAN R, LINEHAN WM, REES RC, HARRIS CC, LIOTTA LA AND STEEG PS. (1991). Somatic allelic deletion of nm23 in human cancer. *Cancer Res.*, **51**, 2490-2493.

LI Y, BOLLAG G, CLARK R, STEVENS J, CONROY L, FULTS D, WARD K, FRIEDMAN E, SAMOWITZ W, ROBERTSON M, BRADLEY P, MCCORMICK F, WHITE R AND CAWTHON R. (1992). Somatic mutations in the neurofibromatosis 1 gene in human tumors. *Cell*, **69**, 275-281.

LINDBLOM A, SKOOG L, ANDERSEN T, ROTSTEIN S, NORDENSKJOLD M AND LARSSON C. (1993). Four separate regions on chromosome 17 show loss of heterozgosity in familial breast carcinomas. *Hum. Genet.*, **91**, 6-12.

MILLER SA, DYKES DD AND POLESKY HF. (1988). A simple salting out procedure for extracting DNA from human nucleated cells. *Nucleic Acids Res.*, **16**, 1215.

MULERIS M, SALMON R-J AND DUTRILLAUX B. (1990). Cytogenetics of colorectal adenocarcinomas. *Cancer Genet. Cytogenet.*, **46**, 143-156.

NAKAMURA Y, BALLARD L, LEPPERT M, O'CONNEL P, LATHROP GM, LALOUEL J-M AND WHITE R. (1988*a*). Isolation and mapping of a polymorphic DNA sequence (pYNZ22) on chromosome 17p. *Nucleic Acids Res.*, **16**, 5707.

NAKAMURA Y, LATHROP M, O'CONNELL P, LEPPERT M, BARKER D, WRIGHT E, SKOLNICK M, KONDOLEON S, LITT M AND LALOUEL JM. (1988*b*). A mapped set of DNA markers for human chromosome 17. *Genomics*, **2**, 302-309.

NAKAMURA Y, HOLM T, GILLILAN S, LEPPERT M, O'CONNELL P, LATHROP GM, LALOUEL J-M AND WHITE R. (1988*c*). Isolation and mapping of a polymorphic DNA sequence (pTHH59) on chromosome 17q. *Nucleic Acids Res.*, **16**, 3598.

NEWLAND RC, CHAPIUS PH AND SMYTH EJ. (1987). The prognostic value of substaging colorectal carcinoma. *Cancer*, **60**, 852-857.

NIH/CEPH COLLABORATIVE MAPPING GROUP. (1992). A comprehensive genetic linkage map of the human genome. *Science*, **258**, 67-86.

SAITO H, INAZAWA J, SAITO S, KASUMI F, KOI S, SAGAE S, KUDO R, SAITO J, NODA K AND NAKAMURA Y. (1993). Detailed deletion mapping of chromosome 17q in ovarian and breast cancers: 2-cM region on 17q21.3 often and commonly deleted in tumors. *Cancer Res.*, **53**, 3382-3385.

SATO T, SAITO H, SWENSEN J, OLIFANT A, WOOD C, DANNER D, SAKAMOTO T, TAKITA K, KASUMI F, MIKI Y, SKOLNICK M AND NAKAMURA Y. (1992). The human prohibitin gene located on chromosome 17q21 is mutated in sporadic breast cancer. *Cancer Res.*, **52**, 1643-1646.

SOLOMON E, VOSS R, HALL V, BODMER WF, JASS JR, JEFFREYS AJ, LUCIBELLO FC, PATEL I AND RIDER SH. (1987). Chromosome 5 allele loss in human colorectal carcinomas. *Nature*, **328**, 616-619.

VARESCO L, CALIGO MA, SIMI P, BLACK DM, NARDINI V, CASARINO L, ROCCHI M, GERRARA G, SOLOMON E AND BEVILACQUA G. (1992). The NM23 gene maps to human chromosome band 17q22 and shows a restriction fragment length polymorphism with BglII. *Genes Chrom. Cancer*, **4**, 84-88.

VOGELSTEIN B, FEARON ER, HAMILTON SR, KERN SE, PREISINGER AC, LEPPERT M, NAKAMURA Y, WHITE R, SMITS AM AND BOS JL. (1988). Genetic alterations during colorectal-tumor development. *N. Engl. J. Med.*, **319**, 525-532.

VOGELSTEIN B, FEARON ER, KERN SE, HAMILTON SR, PREISINGER AC, NAKAMURA Y AND WHITE R. (1989). Allelotype of colorectal carcinomas. *Science*, **244**, 207-211.

WANG L, PATEL V, GHOSH L, CHEN H-C AND BANERJEE S. (1993). Mutation in the nm23 gene is associated with metastasis in colorectal cancer. *Cancer Res.*, **53**, 717-720.

WEINBERG RA. (1991). Tumor suppressor genes. *Science*, **254**, 1138-1146.

YAGUE J, JUAN M, LEONE A, ROMERO M, CARDESA A, VIVES J, STEEG PS AND CAMPO E. (1991). BglII and EcoRI polymorphism of the human nm23-H1 gene (NME1). *Nucleic Acids Res.*, **19**, 6663.

YOUNG JP, SEARLE J, STITZ R, COWEN A, WARD M AND CHENEVIX-TRENCH G. (1992). Loss of heterozygosity at the human RAP1A/Krev-1 locus is a rare event in colorectal tumors. *Cancer Res.*, **52**, 285-289.